# Outbreak of hepatitis A associated with men who have sex with men (MSM), England, July 2016 to January 2017

**DOI:** 10.2807/1560-7917.ES.2017.22.5.30454

**Published:** 2017-02-02

**Authors:** Kazim Beebeejaun, Srilaxmi Degala, Koye Balogun, Ian Simms, Sarah Charlotte Woodhall, Ellen Heinsbroek, Paul David Crook, Ishani Kar-Purkayastha, Juli Treacy, Kate Wedgwood, Kate Jordan, Sema Mandal, Siew Lin Ngui, Michael Edelstein

**Affiliations:** 1 Immunisation, Hepatitis and Blood Safety department, National Infection Service, Public Health England, Colindale, London, United Kingdom; 2 Field Epidemiology Services East Midlands, Public Health England, United Kingdom; 3 HIV and STI department, National Infection Service, Public Health England, Colindale, London, United Kingdom; 4 Field Epidemiology Services London, Public Health England, United Kingdom; 5 Hampshire & Isle of Wight Health Protection Team (South East), Public Health England, United Kingdom; 6 East Midlands Health Protection Team, Public Health England, United Kingdom; 7 South West Health Protection Team, Public Health England, United Kingdom; 8 Virus Reference Department, Public Health England, Colindale, London, United Kingdom

**Keywords:** hepatitis A, outbreaks, MSM, England, Spain, sexually transmitted infections

## Abstract

Between July 2016 and January 2017, 37 confirmed cases of hepatitis A with two unique IA genotype strains primarily among men who have sex with men, were reported across eight areas in England and Northern Ireland. Epidemiological and laboratory investigations indicate that these strains may have been imported several times from Spain, with secondary sexual transmission in the United Kingdom. Local and national public health services are collaborating to control this ongoing outbreak.

Infection with the hepatitis A virus (HAV) is most commonly acquired through ingestion of contaminated food and water, and through faeco-oral contact. In the United Kingdom (UK) hepatitis A is a rare and mainly travel-associated disease, preventable by vaccination [1,2]. Sexually transmitted hepatitis A outbreaks among men who have sex with men (MSM) are well documented [3-6]. We describe an ongoing outbreak in the UK, primarily affecting MSM, caused by two concurrently circulating HAV strains previously not seen in the UK, as well as the intervention strategies that have been instigated to control the outbreak. Cases with the identical strains have been reported in other European countries, prompting the European Centre for Disease Prevention and Control (ECDC) to issue a rapid risk assessment in December 2016 [7].

## Case definition

A confirmed case was defined as a laboratory-confirmed HAV infection with the specific outbreak sequence of either VRD_521_2016 Strain 1 (Event 1) or RIVM-HAV16–090 Strain 2 (Event 2) and symptom onset after 31 June 2016 [7]. A probable case was defined as a laboratory-confirmed HAV infection (not yet sequenced) with symptom onset after 31 June 2016, with contact with a confirmed case and/or who identifies as MSM.

## Outbreak description

**Figure 1:**
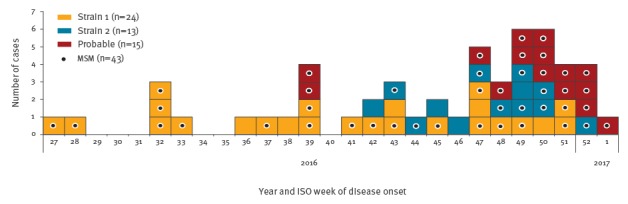
Probable and confirmed cases of hepatitis A among men who have sex with men, England and Northern Ireland, July 2016–January 2017 (n=52)

ISO: International Organization for Standardization; MSM: men who have sex with men.

Between July 2016 and January 2017, 37 confirmed cases with either strain 1 or 2 were detected across England as well as Northern Ireland (Figure 1), of which 28 identified as MSM. Of the 37 cases, 24 were Strain 1 and 13 were Strain 2. In addition, 15 probable cases (all MSM), primarily in London, were identified, and typing results are awaited.

**Figure 2:**
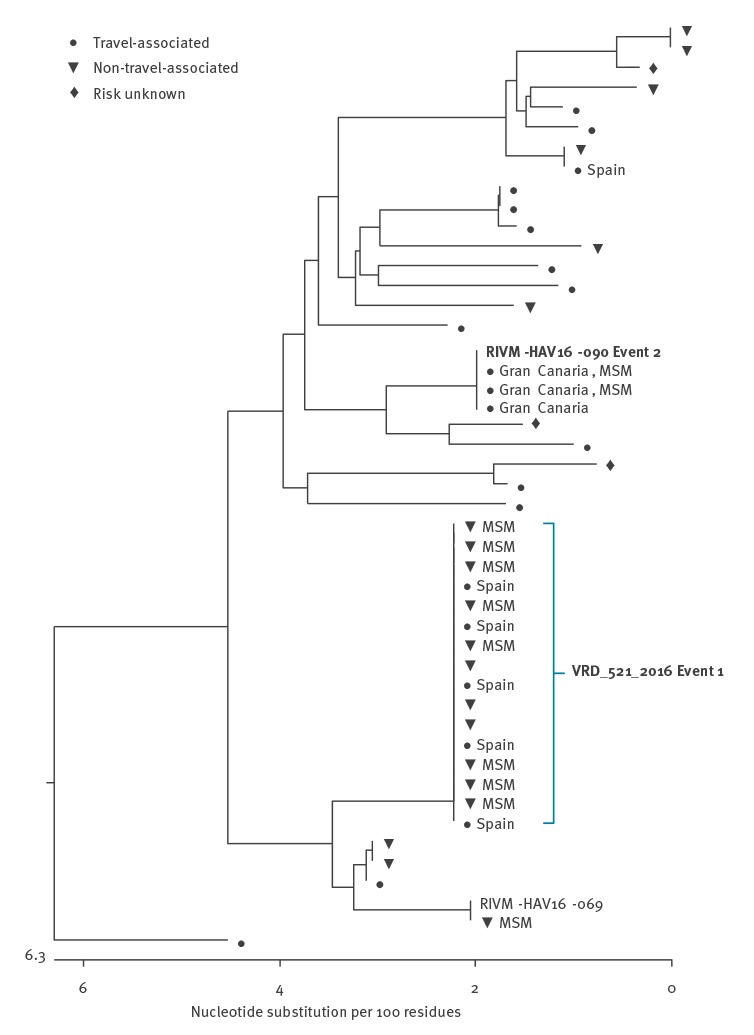
Phylogenetic analysis of virus strains from hepatitis A cases in England and Northern Ireland, July 2016–December 2016

HAV: hepatitis A virus; MSM: men who have sex with men.

Strain 1 was first identified by the Virus Reference Department, Public Health England, London, in July 2016. The sequence had not been seen previously in the UK and phylogenetic analysis (Figure 2) showed a clear relation to sequences derived from travellers returning from Central and South America.

The tree was constructed in MegAlign (DNAstar) using Clustal Key.

**Figure 3:**
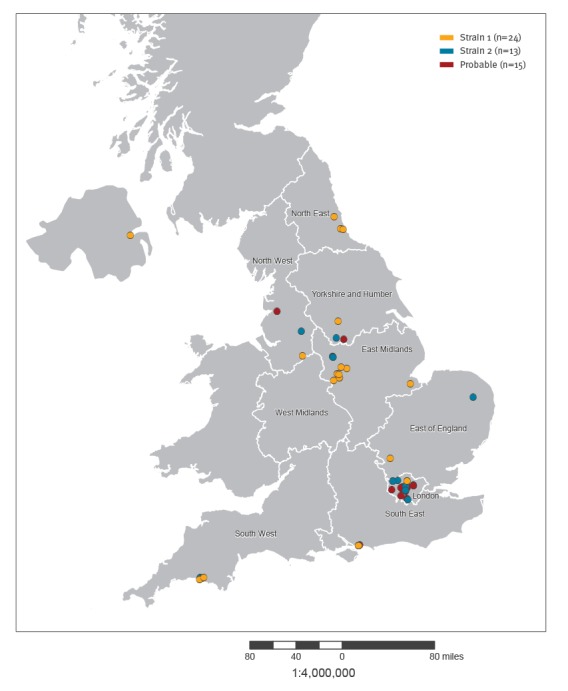
Geographical distribution of hepatitis A cases among men who have sex with men, England and Northern Ireland, July 2016–January 2017 (n=52)

Strain 1 cases were reported in eight geographically distinct areas in England and Northern Ireland (Figure 3).

Contains Ordnance Survey data. Crown copyright and database right 2017. Contains National Statistics data. Crown copyright and database right 2017.

Of 24 Strain 1 cases, 22 were male, median age 35 years (19–63 years), 19 identified as MSM and eight reported travel within the incubation period, seven of which to Spain (Table).

**Table:** Characteristics of hepatitis A cases associated with the outbreak, England and Northern Ireland, July 2016–January 2017 (n=52)

Region	Case status (strain)	Cases (n)	Median age (years)	MSM (n)	Spain	Notable characteristics
**East Midlands**	**Confirmed (Strain 1)**	9	28	6	2	One cluster of three cases of Strain 1 transmitted in a factory through environmental exposure.
	**Confirmed (Strain 2)**	3	55	2	2	
	**Probable**	0	NA	0	0	
	**Total**	**12**	**36**	**8**	**4**	
**South West**	**Confirmed (Strain 1)**	4	45	3	1	One case operated a private meeting place, used by contacts and multiple anonymous men.
	**Confirmed (Strain 2)**	1	NA	0	1	
	**Probable**	0	NA	0	0	
	**Total**	**5**	**46**	**3**	**2**	
**Hampshire**	**Confirmed (Strain 1)**	3	35	3	1	Probable case is index case in this area. This case was diagnosed in Spain but never sequenced. Further spread through household and sexual contacts.
	**Confirmed (Strain 2)**	0	NA	0	0	
	**Probable**	1	NA	1	1	
	**Total**	**4**	**32**	**4**	**2**	
**North East**	**Confirmed (Strain 1)**	3	41	3	1	First identified case with likely importation from Spain. Further spread to two cases through household and sexual transmission.
	**Confirmed (Strain 2)**	0	NA	0	0	
	**Probable**	0	NA	0	0	
	**Total**	**3**	**41**	**3**	**1**	
**London**	**Confirmed (Strain 1)**	2	31	2	0	One Strain 1 case was a sex worker with multiple sexually-transmitted co-infections who reported sex in several gay saunas in London. Three cases reported using apps and websites to meet partners. One Strain 2 case reported 20 sexual contacts within the eight weeks prior to disease onset.
	**Confirmed (Strain 2)**	6	35	4	3	
	**Probable**	12	34	12	1	
	**Total**	**20**	**32**	**18**	**4**	
**Yorkshire and Humber**	**Confirmed (Strain 1)**	1	NA	0	0	All but one case reported travel; three to Spain and to Germany. One Strain 2 case reported sexual contact with multiple partners at a gay sauna in London.
	**Confirmed (Strain 2)**	1	NA	1	1	
	**Probable**	1	NA	1	0	
	**Total**	**3**	**NA**	**2**	**1**	
**North West**	**Confirmed (Strain 1)**	0	NA	0	0	
	**Confirmed (Strain 2)**	1	NA	1	0	
	**Probable**	1	NA	1	0	
	**Total**	**2**	**43**	**2**	**0**	
**East of England**	**Confirmed (Strain 1)**	0	NA	0	0	
	**Confirmed (Strain 2)**	1	NA	1	0	
	**Probable**	0	NA	0	0	
	**Total**	**1**	**NA**	**1**	**0**	
**South Midlands**	**Confirmed (Strain 1)**	1	NA	1	1	
	**Confirmed (Strain 2)**	0	NA	0	0	
	**Probable**	0	NA	0	0	
	**Total**	**1**	**NA**	**1**	**1**	
**Belfast**	**Confirmed (Strain 1)**	1	NA	1	1	
	**Confirmed (Strain 2)**	0	NA	0	0	
	**Probable**	0	NA	0	0	
	**Total**	**1**	**NA**	**1**	**1**	
**Grand total**		**52**	**36**	**43**	**16**	

MSM: men who have sex with men; NA: not applicable.

Strain 2 was first notified through the European Union Early Warning and Response System (EWRS) message from the Netherlands in October 2016 related to two MSM cases at EuroPride 2016, which took place in Amsterdam in July/August 2016. This genotype sequence was detected in 13 cases across six regions in England between November 2016 and January 2017 (Figure 3). Of the 13 cases, 12 were male, median age 39 years (range: 29–78), nine identified as MSM and 11 travelled during the incubation period, of which seven to Spain and two to Germany (Table). Of note, Strain 2 has mainly been reported in MSM in London to date. Characteristics of concern among cases were noted, including infection in a sex worker with multiple partners, co-infection with sexually transmitted infections (STIs) and use of sex-on-site premises and apps (Grindr, Recon) (Table).

## Control measures

Public Health England (PHE) declared a national incident in December 2016. Local and national laboratory, epidemiology and health protection teams contributed to the response, which comprised: (i) enhanced surveillance for MSM-associated cases through an adapted questionnaire [8], (ii) a joint letter with the British Association for Sexual Health and HIV (BASHH) to all members alerting them to the outbreak and recommending vaccination of at-risk MSM in outbreak areas, according to national guidelines [9,10], testing cases for other STIs and partner notification, (iii) disease information and targeted hygiene advice to the public through the National Health Service web portal [11], (iv) liaising with lesbian, gay, bisexual, and transgender (LGBT) and sexual health charities, gay-dating apps and gay venues to raise awareness through social media and health promotion visuals, and (v) giving post-exposure prophylaxis to household and sexual contacts.

## Discussion

As at 24 January 2017, 37 HAV infections with two sequences have been identified in eight UK areas, mostly among MSM (median age: 35 years; range: 19-56). HAV infection is most commonly acquired through contaminated food or water. In this outbreak however, epidemiological and laboratory investigations suggest multiple importations from several regions of Spain with secondary sexual transmission within the MSM population in the UK, as nine of the confirmed MSM cases reported travelling to Spain during the incubation period. Ireland, Sweden, Luxembourg and Germany have reported hepatitis A cases with identical viral sequences, some with history of travel to Spain during the incubation period. Spain has reported an increase in male HAV infections, but no further details were available [7]. This outbreak highlights the key role sequencing can play in outbreak detection, as well as the added value of a common European platform to share epidemiological and virological information.

While the two concurrently circulating strains are virologically distinct, the public health response is intended to address both. Although it has not been possible to establish epidemiological links between all cases within geographical clusters, it is likely that cases are related either through undisclosed sexual contacts or other routes since neither strain is commonly circulating in England. These missing epidemiological links are not unexpected when trying to capture sexual history via short questionnaires, particularly since some cases reported anonymous sex with multiple partners. However, the questionnaires revealed sex-on-premises venues (saunas, clubs) and social networking (dating apps) as potential drivers of the outbreak. While these findings can help focus interventions, they are of particular concern in areas with large, active MSM populations, such as London, where several of the recent cases have been reported.

This outbreak also highlights the need for HAV awareness among MSM and sexual health professionals and the need for health promotion materials that focus on both infection and vaccination. Innovative and evaluated communication strategies with targeted messaging through social media, apps and venues also need to be readily available to public health agencies.

Hepatitis A vaccination for MSM in England is currently a risk-based recommendation [9,10]. For the purpose of this investigation, the vaccination status of the cases was not included in the analysis. While some may advocate for a universal MSM vaccination policy, it may not be cost-effective or affordable for local governments who commission sexual health services. Vaccine availability also needs to be taken into account as it may impact the ability to vaccinate a large number of individuals in a short timeframe. Enhanced surveillance for HAV in MSM will allow monitoring of the evolving outbreak as well as evaluating intervention impact, and gain a better understanding of HAV transmission in this population.

## References

[1] Public Health England. Laboratory reports of hepatitis A infection, and hepatitis C: 2015. Health Protection Report. 2016;10. [Accessed 16 Dec 2016]. Available from: https://www.gov.uk/government/publications/laboratory-reports-of-hepatitis-a-and-c-2015

[2] European Centre for Disease Prevention and Control (ECDC). Hepatitis A virus in the EU/EEA, 1975-2014. ECDC technical report. Stockholm: ECDC. 2016. Available from: http://ecdc.europa.eu/en/publications/Publications/hepatitis-a-virus-EU-EEA-1975-2014.pdf

[3] Bell A Ncube F Hansell A Davison KL Young Y Gilson R An outbreak of hepatitis A among young men associated with having sex in public venues. Commun Dis Public Health. 2001;4(3):163-70. 11732354

[4] Mindel A Tedder R Hepatitis A in homosexuals. Br Med J (Clin Res Ed). 1981;282(6277):1666. . 10.1136/bmj.282.6277.1666 6786425 PMC1505651

[5] Sfetcu O Irvine N Ngui SL Emerson C McCaughey C Donaghy P Hepatitis A outbreak predominantly affecting men who have sex with men in Northern Ireland, October 2008 to July 2009. Euro Surveill. 2011;16(9):19808. Available from: http://www.eurosurveillance.org/ViewArticle.aspx?ArticleId=19808 21392487

[6] Stene-Johansen K Tjon G Schreier E Bremer V Bruisten S Ngui SL Molecular epidemiological studies show that hepatitis A virus is endemic among active homosexual men in Europe. J Med Virol. 2007;79(4):356-65. . 10.1002/jmv.20781 17311331

[7] European Centre for Disease Prevention and Control (ECDC). Hepatitis A outbreaks in the EU/EEA mostly affecting men who have sex with men. Stockholm: ECDC. 19 Dec 2016. Available from: http://ecdc.europa.eu/en/publications/Publications/13-12-2016-RRA-Hepatitis%20A-United%20Kingdom.pdf

[8] Public Health England (PHE). Hepatitis A: case questionnaire. London: PHE. 2016. [Accessed 16 Dec 2016]. Available from: https://www.gov.uk/government/publications/hepatitis-a-case-questionnaire

[9] Public Health England (PHE). Green Book, Immunisation against infectious disease: Chapter 17, Hepatitis A. London: PHE. [Accessed 16 Dec 2016]. Available from: https://www.gov.uk/government/publications/hepatitis-a-the-green-book-chapter-17

[10] Brook G Bhagani S Kulasegaram R Torkington A Mutimer D Hodges E Clinical Effectiveness Group British Association for Sexual Health and HIV United Kingdom National Guideline on the Management of the viral hepatitides A, B and C 2015. Int J STD AIDS. 2016;27(7):501-25. 10.1177/0956462415624250 26745988

[11] NHS Choices. Sexual health for gay and bisexual men - Live Well - NHS Choices. Hepatitis A. [Accessed 16 Dec 2016]. Available from: http://www.nhs.uk/Livewell/LGBhealth/Pages/Sexualhealthgaymen.aspx#hepa

